# Supplementary material for: Photoreceptor disc incisures form as an adaptive mechanism ensuring the completion of disc enclosure
Source: bioRxiv. 2023 Apr 7:2023.04.06.535932. Preprint. [Version 1] doi: 10.1101/2023.04.06.535932 (PMC10104153; doi:10.1101/2023.04.06.535932)
Supplement: Supplement 1 [file NIHPP2023.04.06.535932v1-supplement-1.pdf]

bioRxiv preprint doi: https://doi.org/10.1101/2023.04.06.535932; this version posted April 7, 2023. The copyright holder for this preprint (which was not certified by peer review) is the author/funder, who has granted bioRxiv a license to display the preprint in perpetuity. It is made available under aCC-BY 4.0 International license.

Supplementary Figures

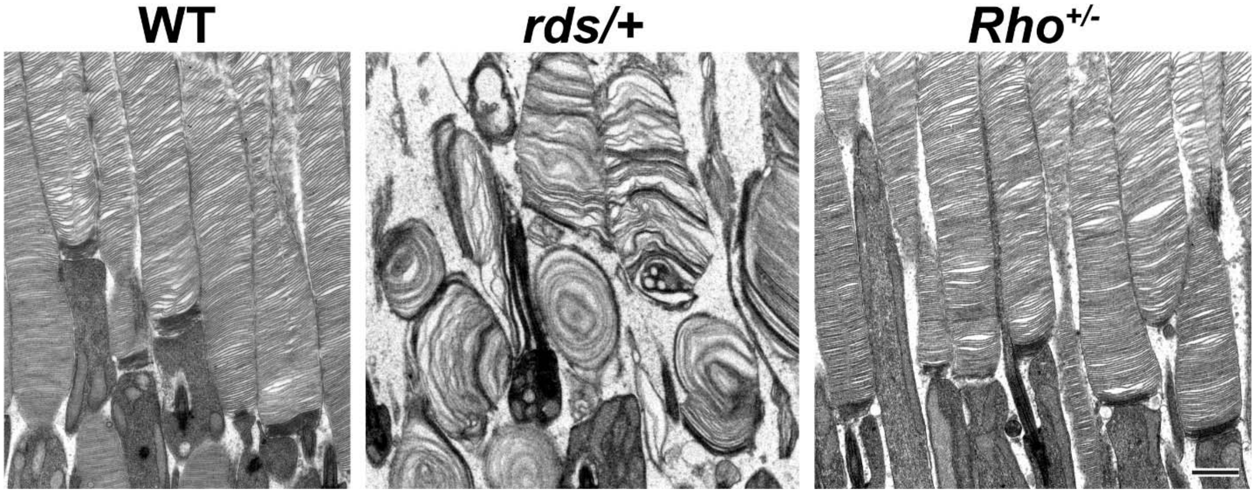

Figure S1. Reduction in the level of peripherin-2 but not rhodopsin causes gross abnormalities in outer segment structure

Representative TEM images of longitudinally sectioned WT, *rds/+* and *Rho*$^{+/-}$ mouse retinas. Scale bar: 1 µm.

bioRxiv preprint doi: https://doi.org/10.1101/2023.04.06.535932; this version posted April 7, 2023. The copyright holder for this preprint (which was not certified by peer review) is the author/funder, who has granted bioRxiv a license to display the preprint in perpetuity. It is made available under aCC-BY 4.0 International license.

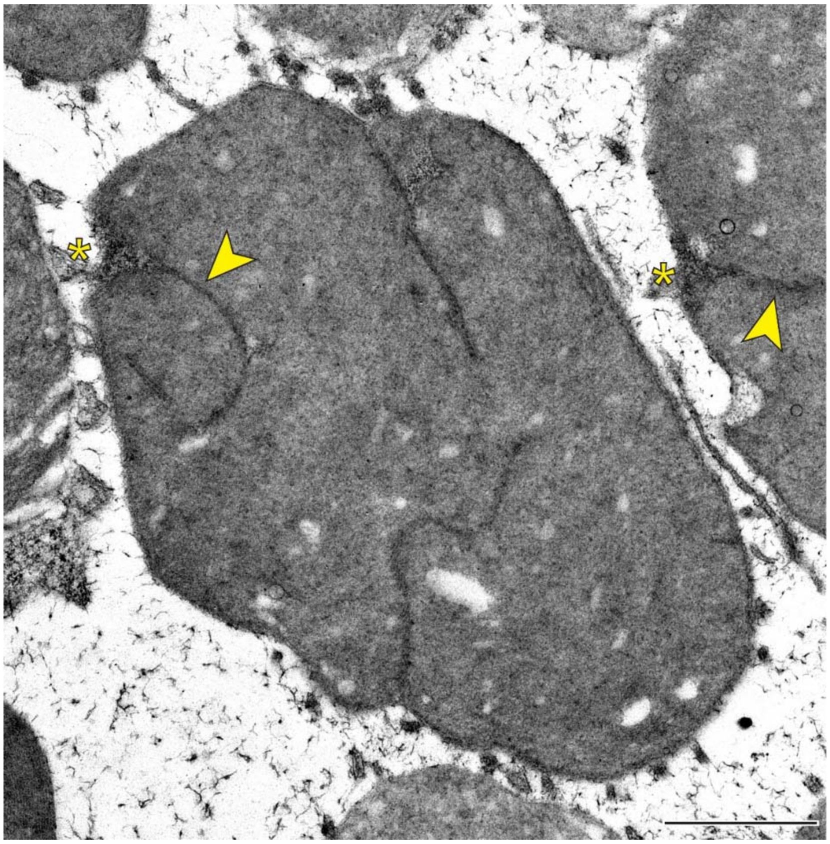

Figure S2. One incisure in frog rod discs is aligned with the ciliary axoneme

Representative TEM image of a tangentially sectioned WT frog retina. Yellow asterisks indicate the ciliary axoneme; yellow arrowheads point to incisures. Scale bar: 1 µm.

bioRxiv preprint doi: https://doi.org/10.1101/2023.04.06.535932; this version posted April 7, 2023. The copyright holder for this preprint (which was not certified by peer review) is the author/funder, who has granted bioRxiv a license to display the preprint in perpetuity. It is made available under aCC-BY 4.0 International license.

Supplementary Table

Table S1. Quantification of molar ratios between rhodopsin, peripherin-2 and ROM1 in mouse outer segments – raw data

Movie Legends

Movie 1. Tomogram associated with Figure 3.

Movie 2. Tomogram associated with Figure 4A.

Movie 3. Tomogram associated with Figure 4B.

Movie 4. Tomogram associated with Figure 4C.

Movie 5. Tomogram associated with Figure 4D.

Movie 6. Reconstructed tomogram of a basal body nucleating the ciliary axoneme. Shown is a 420 nm fragment of a 750 nm-thick retinal section. Tomogram pixel size is 0.7 nm.

Movie 7. Tomogram associated with Figure 5.
